# Effect of white-matter lesions on the risk of periprocedural stroke after carotid artery stenting versus endarterectomy in the International Carotid Stenting Study (ICSS): a prespecified analysis of data from a randomised trial

**DOI:** 10.1016/S1474-4422(13)70135-2

**Published:** 2013-09

**Authors:** Jörg Ederle, Indran Davagnanam, H Bart van der Worp, Graham S Venables, Philippe A Lyrer, Roland L Featherstone, Martin M Brown, H Rolf Jäger, on behalf of the ICSS investigators

**Affiliations:** a Stroke Research Group, Department of Brain Repair and Rehabilitation, UCL Institute of Neurology, Queen Square, London, UK; b Lysholm Department of Neuroradiology, UCL Institute of Neurology, Queen Square, London, UK; c Utrecht Stroke Center, Departments of Neurology, Rudolf Magnus Institute of Neuroscience, University Medical Center Utrecht, the Netherlands; d Neurology Department, Royal Hallamshire Hospital, Sheffield, UK; e Department of Neurology, University Hospital Basel, Basel, Switzerland

## Abstract

**Background:**

Findings from randomised trials have shown a higher early risk of stroke after carotid artery stenting than after carotid endarterectomy. We assessed whether white-matter lesions affect the perioperative risk of stroke in patients treated with carotid artery stenting versus carotid endarterectomy.

**Methods:**

Patients with symptomatic carotid artery stenosis included in the International Carotid Stenting Study (ICSS) were randomly allocated to receive carotid artery stenting or carotid endarterectomy. Copies of baseline brain imaging were analysed by two investigators, who were masked to treatment, for the severity of white-matter lesions using the age-related white-matter changes (ARWMC) score. Randomisation was done with a computer-generated sequence (1:1). Patients were divided into two groups using the median ARWMC. We analysed the risk of stroke within 30 days of revascularisation using a per-protocol analysis. ICSS is registered with controlled-trials.com, number ISRCTN 25337470.

**Findings:**

1036 patients (536 randomly allocated to carotid artery stenting, 500 to carotid endarterectomy) had baseline imaging available. Median ARWMC score was 7, and patients were dichotomised into those with a score of 7 or more and those with a score of less than 7. In patients treated with carotid artery stenting, those with an ARWMC score of 7 or more had an increased risk of stroke compared with those with a score of less than 7 (HR for any stroke 2·76, 95% CI 1·17–6·51; p=0·021; HR for non-disabling stroke 3·00, 1·10–8·36; p=0·031), but we did not see a similar association in patients treated with carotid endarterectomy (HR for any stroke 1·18, 0·40–3·55; p=0·76; HR for disabling or fatal stroke 1·41, 0·38–5·26; p=0·607). Carotid artery stenting was associated with a higher risk of stroke compared with carotid endarterectomy in patients with an ARWMC score of 7 or more (HR for any stroke 2·98, 1·29–6·93; p=0·011; HR for non-disabling stroke 6·34, 1·45–27·71; p=0·014), but there was no risk difference in patients with an ARWMC score of less than 7.

**Interpretation:**

The presence of white-matter lesions on brain imaging should be taken into account when selecting patients for carotid revascularisation. Carotid artery stenting should be avoided in patients with more extensive white-matter lesions, but might be an acceptable alternative to carotid endarterectomy in patients with less extensive lesions.

**Funding:**

Medical Research Council, the Stroke Association, Sanofi-Synthélabo, the European Union Research Framework Programme 5.

## Background

Findings from randomised controlled trials comparing carotid artery stenting with carotid endarterectomy have consistently shown that, in patients with symptomatic carotid stenosis, carotid artery stenting is associated with a higher risk of periprocedural stroke or death than carotid endarterectomy.^1^, ^2^, ^3^, ^4^ Findings from a systematic review of 11 randomised controlled trials showed a 70% increase in the risk of stenting compared with endarterectomy in terms of the occurrence of any stroke or death between randomisation and 30 days after treatment in symptomatic patients (odds ratio 1·72, 95% CI 1·29–2·31).^5^ A meta-analysis of individual patient data from the three European trials^6^ showed that the difference in early stroke or death rates favouring carotid endarterectomy was attributable to the treatment of patients older than 70 years, and that in patients younger than 70 years there was no difference in risk.^5^ Similar findings were reported in the North American trial.^4^ No other subgroup variable has consistently been shown to affect the difference in risk favouring carotid endarterectomy. However, findings from the trials also confirmed that carotid artery stenting avoids some of the other complications of carotid endarterectomy, especially cranial nerve injury and myocardial infarction, and has a lower rate of wound haematoma.^5^ Therefore, a better understanding of risk factors for procedural stroke would help inform clinicians as to the best treatment option for their patients.

That carotid artery stenting, and to a lesser extent carotid endarterectomy, can cause embolisation of thrombus or plaque debris from the carotid plaque during the revascularisation procedure is well recognised. We hypothesised that severe cerebral white-matter damage might render patients more vulnerable to the effects of embolisation and therefore more likely to manifest stroke; furthermore, this increased risk might differ between carotid artery stenting and carotid endarterectomy.

Abnormalities in white matter are a common finding on CT and MRI scans of the brain.^7^, ^8^, ^9^, ^10^, ^11^, ^12^, ^13^ In 1987, Hachinski and colleagues proposed the term leukoaraiosis as a purely descriptive term for the presence of patchy diffuse changes in the periventricular cerebral white matter.^12^ Nowadays, the term white-matter lesions is more commonly used for the same findings. In most patients, these lesions are likely to be the consequence of degenerative changes in the deep perforating arterioles. In patients with stroke, the reported prevalence of white-matter lesions is between 4% and 44% on CT and up to 100% on MRI.^7^, ^9^, ^10^ Risk factors for the development of white-matter lesions include age and hypertension.

Leukoaraiosis was associated with a higher perioperative risk of stroke or death in patients assigned to carotid endarterectomy in the North American Carotid Endarterectomy Trial (NASCET).^14^ Patients with widespread white-matter changes allocated to the best medical management group also had an increased risk of stroke or death. To the best of our knowledge, the effect of white-matter lesions on the procedural risk of stroke and death in carotid stenting has hitherto not been investigated.

We therefore investigated the effect of leukoaraiosis on the risk of procedural complications in a large group of patients with recently symptomatic carotid disease randomised in the International Carotid Stenting Study.

## Methods

### Patients

The International Carotid Stenting Study (ICSS) is an international multicentre randomised clinical trial comparing carotid stenting and endarterectomy in patients with symptomatic carotid atheromatous stenosis. The ICSS protocol and the results of an interim analysis of the short-term outcome events within 120 days of randomisation are available elsewhere.^3^, ^15^ Briefly, ICSS included patients older than 40 years with atherosclerotic carotid artery stenosis measuring 50% or more which had been symptomatic within the previous 12 months. Patients were excluded if they had major stroke with no useful recovery of function, were not suitable for endarterectomy for anatomical or medical reasons, or if the stenosis was considered unsuitable for stenting. For patients to be randomly assigned to treatment, the investigators had to be uncertain about which of the two treatment options would be best for the patient.

All patients participating in ICSS provided written informed consent. Brain imaging by CT or MRI was needed before revascularisation. We included in this study of white-matter lesions all patients enrolled in ICSS in whom copies of the baseline CT or MRI done before carotid stenting or endarterectomy were available. Patients were excluded if no baseline brain imaging was available or if the quality of the images was poor.

ICSS was approved by the Northwest Multicentre Research Ethics Committee in the UK and participating centres had to obtain site-specific approval from their local ethics committee.

### Randomisation and masking

Patients in the ICSS were randomly assigned in a one-to-one ratio to receive carotid artery stenting or carotid endarterectomy. Randomisation was by telephone call or fax to a central computerised service and was stratified by centre with minimisation for sex, age, contralateral occlusion, and side of the randomised artery. Patients and investigators were not masked to treatment assignment. Patients were followed-up at 1 month after revascularisation by independent clinicians not directly involved in delivering the randomised treatment. Adjudication of the main outcome events was done by a masked independent outcome event committee.

### Procedures

We defined stroke as an acute disturbance of focal neurological function with symptoms lasting more than 24 h, resulting from intracranial vascular disturbance. We included visual loss resulting from embolic or haemodynamic retinal ischaemia lasting more than 24 h. We classified events leading to a modified Rankin score of 3 or greater for more than 30 days after onset as disabling; the remaining events were classified as non-disabling. Events leading to death within 30 days after onset were classified as fatal. We defined a procedural event as one occurring within 30 days of treatment.

Anonymised diagnostic brain imaging before treatment was analysed at the central trial office (University College London, Institute of Neurology, London, UK). Two investigators (JE and ID) trained in the analysis of white-matter lesions and masked to treatment and clinical outcome rated all images by consensus, using the age-related white-matter changes (ARWMC) score first introduced and validated for use in CT and MRI by Wahlund and colleagues.^16^ White-matter lesions in the cerebral hemispheres and the brainstem were identified as poorly defined hyperintensities 5 mm or larger in diameter on T2-weighted or FLAIR MRI images, or areas of poorly defined hypodensity of 5 mm or larger in diameter on CT. We rated changes in the basal ganglia in the same manner. We rated the extent of white-matter disease on a 4-point scale in different brain regions on T2-weighted and FLAIR MRI images or on CT images: 0=no lesions (including symmetrical, well defined caps or bands); 1=focal lesions; 2=beginning confluence of lesions; 3=diffuse involvement of the entire region, with or without involvement of U fibres.

We assessed the scans in five brain regions in each patient separately in the left and right hemispheres: the frontal area (the frontal lobe anterior to the central sulcus); the parieto-occipital area (the parietal and occipital lobes in combination); the temporal area (the temporal lobe, with a line from the posterior part of the Sylvian fissure to the trigones of the lateral ventricles separating this area from the parieto-occipital area); the infratentorial area (which included the brainstem and cerebellum); and the basal ganglia region (which included the striatum, globus pallidus, thalamus, external capsules, and insula).

The total ARWMC score was then obtained by adding the scores for each brain region. Thus, the total ARWMC score ranged from 0 to 30. In addition to the rating of white-matter lesions, we recorded the presence of established cerebral infarcts. We did not record the location of these infarcts.

As expected in an international multicentre trial, various scanners and scanning protocols were used. We rated images available on film using standardised diagnostic grade light boxes. Digital imaging was reviewed using an open-source DICOM viewer software (OsiriX version 3.x). For patients in whom both CT and MRI had been done, we chose MRI for rating white-matter changes.

### Statistical analysis

We tested two hypotheses. The first was that, compared with less extensive white-matter lesions, more extensive white-matter lesions are associated with an increased risk of procedural stroke after treatment of carotid artery disease by either carotid artery stenting or carotid endarterectomy. The second was that the increase in procedural risk would differ in patients treated by carotid artery stenting compared with carotid endarterectomy. The plan to analyse imaging findings in relation to procedural risk was prespecified in the trial protocol. The details of the methods and statistical analyses described here were decided before any results were analysed.

The analyses were done for the following outcome measures: any stroke, non-disabling stroke, and disabling or fatal stroke. Data were analysed per protocol—ie, the analysis included only patients who received the allocated treatment as their first ipsilateral revascularisation procedure. Patients who received the alternative revascularisation procedure as their first treatment (cross-overs), or who received no revascularisation treatment, were excluded from this analysis. All outcome events occurring within 30 days after initiation of the first allocated treatment were included.

Patients were dichotomised into two groups at the median value for the age-related white-matter changes score. The median was chosen as the cut-point for the analysis before doing any analyses to ensure equal-sized groups of patients for the comparisons and to avoid the risk of the bias introduced by selecting an optimum cut point after initial analysis of the data.^17^ The baseline characteristics in the two treatment groups were then compared according to whether their ARWMC score was lower than 7 versus 7 or greater using Pearson's χ^2^ test or analysis of variance. We compared the 30-day risk of the various outcome events between the two treatment groups using a Cox proportional hazards model. We estimated the cumulative incidences of the different outcome measures using Kaplan-Meier analysis and compared them using log-rank tests stratified by treatment with ARWMC score dichotomised at the median. All analyses were adjusted for age, sex, and vascular risk factors. We used SPSS (version 21) for all statistical analyses.

ICSS is registered with controlled-trials.com, number ISRCTN 25337470.

### Role of the funding source

The sponsors of the study had no role in study design, data collection, data analysis, data interpretation, or writing of the report. The corresponding author had full access to all the data in the study and had final responsibility for the decision to submit for publication.

## Results

**Figure 1:**
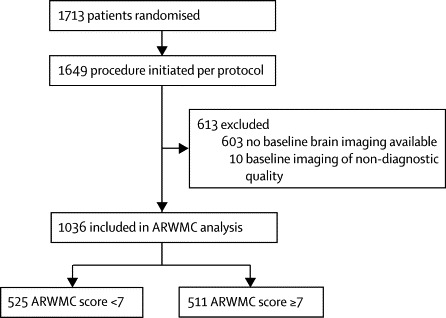
Study profile ARWMC=age-related white-matter changes.

High-quality baseline brain imaging was available from 1036 (61%) of 1649 patients enrolled in ICSS between May, 2001, and October, 2008, in whom the allocated treatment was initiated as the first ipsilateral revascularisation procedure within 30 days of randomisation (figure 1). Of these patients, 536 were allocated to treatment with carotid artery stenting and 500 were allocated to treatment with carotid endarterectomy. CT was analysed in 566 (55%) patients and MRI in 470 (45%) patients. Baseline characteristics were much the same between included patients and those who were excluded because of missing or inadequate imaging (data not shown).

The median ARWMC score at baseline was 7; patients with an ARWMC score of 7 or more were, on average, older and less likely to have had a CT scan only than those with an ARWMC score of less than 7 (table). Hypertension, hypercholesterolaemia, current smoking, and the presence of cerebral infarcts on baseline imaging were associated with a higher ARWMC score. We detected no difference in the proportion of patients receiving carotid artery stenting compared with carotid endarterectomy in the two groups, or in baseline characteristics between the two treatment groups (carotid stenting *vs* endarterectomy).

**Table:** Baseline characteristics

		**Carotid artery stenting (n=536)**		**Carotid endarterectomy (n=500)**		**p values**
		ARWMC score <7 (n=269)	ARWMC score ≥7 (n=267)	ARWMC score <7 (n=256)	ARWMC score ≥7 (n=244)	
CT scan		164 (61%)	121 (45%)	165 (65%)	116 (48%)	<0·0001
Age (mean [SD]; years)		68 (10)	73 (8)	66 (9)	74 (8)	<0·0001
Women		68 (25%)	86 (32%)	75 (29%)	75 (31%)	0·330
Vascular risk factors						
	Cerebral infarct on baseline scan	120 (45%)	153 (57%)	110 (43%)	139 (57%)	0·0003
	Hypertension	181 (68%)	199 (76%)	164 (64%)	180 (75%)	0·009
	Systolic blood pressure (mean [SD]; mm Hg)	145 (25)	151 (25)	146 (23)	148 (24)	0·024
	Diastolic blood pressure (mean [SD]; mm Hg)	80 (12)	80 (13)	79 (12)	79 (13)	0·716
	Diabetes	61 (23%)	58 (26%)	51 (20%)	51 (21%)	0·861
	Treated hyperlipidaemia	168 (63%)	165 (63%)	169 (66%)	154 (64%)	0·869
	Cholesterol (mean [SD]; mmol/L)	4·9 (1·3)	4·8 (1·2)	5·2 (1·4)	4·8 (1·3)	0·008
	Current smoker	73 (27%)	46 (18%)	65 (26%)	41 (17%)	0·005
	Past smoker	117 (44%)	145 (56%)	135 (53%)	123 (51%)	0·051
	Peripheral vascular disease	46 (17%)	45 (17%)	35 (14%)	42 (17%)	0·614
Extent of symptomatic carotid stenosis*						0·581
	50–69%	37 (14%)	31 (12%)	29 (11%)	24 (10%)	
	70–99%	232 (86%)	236 (88%)	227 (89%)	220 (90%)	
Extent of contralateral stenosis*						0·130
	<50%	190 (71%)	170 (64%)	172 (68%)	153 (63%)	
	50–69%	34 (13%)	43 (16%)	40 (16%)	41 (17%)	
	70–99%	24 (9%)	40 (15%)	33 (13%)	39 (16%)	
	Occluded	20 (8%)	11 (4%)	10 (4%)	10 (4%)	
Most recent ipsilateral event†						0·336
	Amaurosis fugax	40 (15%)	42 (16%)	45 (18%)	34 (14%)	
	Transient ischaemic attack	100 (37%)	72 (27%)	80 (31%)	89 (37%)	
	Retinal infarct	8 (3%)	5 (2%)	5 (2%)	4 (2%)	
	Ischaemic hemispheric stroke	119 (44%)	142 (53%)	124 (49%)	114 (47%)	
	Unknown	2 (1%)	6 (2%)	2 (1%)	3 (1%)	
Multiple ipsilateral symptoms before randomisation		120 (45%)	97 (36%)	93 (37%)	99 (41%)	0·154
Ipsilateral stroke before most recent ipsilateral event		42 (16%)	48 (18%)	29 (11%)	39 (16%)	0·194

Data are n (%) unless otherwise indicated. ARWMC=age-related white-matter changes.

* Extent of stenosis measured by the method used in the North American Carotid Endarterectomy Trial^10^ at randomising centre.

† If two events were reported on the same day, we counted the more serious of the two (stroke>retinal infarct>transient ischaemic attack>amaurosis fugax).

In patients in the stenting group, we detected an increase in the risk of stroke in patients with an ARWMC score of 7 or more compared with those with an ARWMC score of less than 7. The hazard ratio (HR) for any stroke was 2·76 (95% CI 1·17–6·51; p=0·021) and the hazard ratio for non-disabling stroke was 3·00 (1·10–8·36; p=0·031). There was a similar proportional increase in the risk of disabling or fatal stroke with ARWMC score of 7 or more in patients who underwent carotid artery stenting, but the number of events available for analysis was smaller than available for the other stroke outcome clusters and the difference was not statistically significant (HR 2·88, 0·61–13·6; p=0·181). By contrast with these findings, in the carotid endarterectomy group, there was no difference in the risk of stroke between patients with a high versus low ARWMC score (HR for any stroke 1·18, 0·40–3·55; p=0·76; HR for non-disabling stroke 1·11, 0·14–8·62; p=0·924; HR for disabling or fatal stroke 1·41, 0·38–5·26, p=0·607).

**Figure 2:**
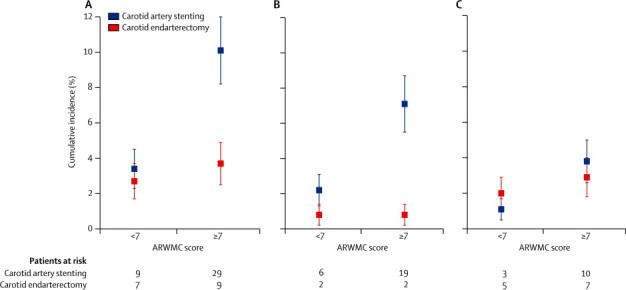
30-day cumulative incidence of stroke by severity of white-matter lesions (A) any stroke, (B) non-disabling stroke, (C) fatal or disabling stroke. ARWMC=age-related white-matter changes. Error bars are SDs.

In patients with an ARWMC score of less than 7, we detected no difference in the risk of any of the stroke outcome clusters between the stenting and endarterectomy groups (figure 2). By contrast, in patients with an ARWMC score of 7 or more, carotid artery stenting was associated with a higher risk of any stroke (HR 2·98, 1·29–6·93; p=0·011) and non-disabling stroke (6·34, 1·45–27·71; p=0·014) compared with carotid endarterectomy. The risk of fatal or disabling stroke in patients with an ARWMC score of 7 or more was not statistically significant between the two treatment groups (HR 1·56; 0·52–4·65; p=0·428).

## Discussion

Our results show that, in patients with symptomatic internal carotid artery stenosis, an increased severity of white-matter damage on CT or MRI is associated with an increased risk of procedural stroke. This increase in the risk of stroke seems to be restricted to patients treated with carotid artery stenting rather than with carotid endarterectomy. Our findings also show that the risk of stenting strongly depends on the severity of white-matter damage: the risk of stroke was similar between the two treatment groups in patients with less extensive white-matter lesions (ARWMC score <7), but much higher for carotid artery stenting than for carotid endarterectomy in patients with more extensive white-matter lesions (ARWMC score ≥7).

We detected no statistically significant increase in the risk of a procedural outcome event with more severe ARWMC in the surgery group. This finding is in contrast with findings from a similar study done in patients in North American Symptomatic Carotid Endarterectomy Trial (NASCET),^14^ which showed a near tripling in the rate of perioperative stroke or death in patients with widespread white-matter changes defined as involving an entire region from the lateral ventricle to the cortex and a doubling of this risk in patients with white-matter changes defined as restricted to the region adjoining the ventricles. However, the fact that the 30-day rate of stroke or death after carotid endarterectomy was 6·7% in NASCET, compared with only 3·4% in ICSS should be borne in mind.^3^, ^18^ By contrast with the findings from NASCET, that we did not detect a significant increase in the periprocedural stroke risk in surgically treated patients with more severe white-matter disease is likely to reflect the improved outcome of surgery over the years.

Findings from a previous ICSS MRI substudy^19^ showed that there is a much higher rate of small emboli detected on DWI MRI after carotid artery stenting than after carotid endarterectomy. One possible explanation for our findings is that a given number of emboli resulting from carotid artery stenting could be more likely to result in symptoms of stroke if pre-existing white-matter damage has reduced cerebrovascular reserve. White-matter changes are thought to be caused at least in part by chronic hypoperfusion, as the result of small and large vessel atherosclerotic disease that leads to chronic white-matter hypoperfusion and changes in the blood–brain barrier.^9^, ^20^, ^21^, ^22^, ^23^, ^24^ White-matter lesions were associated with an increased risk of stroke in the present study and are a risk factor for first-ever and recurrent stroke in large population-based studies.^25^, ^26^, ^27^ A patient with chronic ischaemia of the brain might be more likely to develop infarction after subsequent ischaemic events.^28^ Findings from a study using arterial spin-labelling MR perfusion showed that patients with diffuse confluent white-matter hyperintensities had about 20% lower cerebral blood flow measurements than patients with punctiform or beginning confluent white-matter hyperintensities.^29^ The investigators detected not only a reduction of subcortical white matter and deep-grey matter but also of cortical grey matter and global cerebral blood flow values in patients with diffuse confluent white-matter hyperintensities; the investigators regarded these findings as evidence that such patients have a generalised cerebrovascular disease process rather than one confined to lesions. In keeping with this suggestion, in a series of 70 patients with severe symptomatic carotid stenosis undergoing carotid endarterectomy, the volume of white-matter lesions was associated with the need for intraoperative shunting during carotid endarterectomy, an index of impaired cerebral haemodynamics.^30^ We hypothesise, therefore, that the patients in our study with more extensive white-matter lesions did not have the cerebrovascular reserve and resilience to cope with further ischaemic events.

Another possible explanation for our findings is that patients with more extensive white-matter lesions are more likely to have unstable plaque, making them more vulnerable to embolisation during stent insertion. One study of 57 patients having carotid endarterectomy for symptomatic carotid stenosis reported that the number of white-matter lesions before surgery was associated with the histological finding of unstable plaque.^31^ Evidence also exists that patients with white-matter lesions have a prothrombotic state and endothelial dysfunction that might make them more vulnerable to embolisation during carotid artery stenting.^32^, ^33^

Another possibility is that instead of being a risk factor in its own right, the severity of white-matter disease identified on brain imaging is a marker of the overall effects of individual cardiovascular risk factors.^13^ In their meta-analysis of the data pooled from the three European-based randomised trials, Bonati and colleagues^6^ noted that carotid stenting had a higher procedural risk than endarterectomy only in patients older than 70 years. A meta-analysis of all the published trials has confirmed these findings.^5^ Bonati and colleagues^6^ argued that this association with age might be due to an increased atherosclerotic burden and tortuosity of the aortic arch, with age putting the patients at increased risk of dislodgement of plaque debris. Although these arguments might explain part of the association of risk of carotid artery stenting with increasing age, our findings show an association between the severity of white-matter damage and risk of stroke after correction for age, suggesting that increasing age is not the only explanation for the increased risk.

Several patients randomly allocated in ICSS were excluded from analysis because the original baseline brain images were not available or the images were of inadequate quality for analysis. However, the baseline characteristics of the included patients were not different to those excluded from the study and were well matched between the treatment groups; thus selection bias is unlikely. Nevertheless, the sample size and number of outcome events in each group were smaller than in the entire ICSS cohort, limiting the power of the comparison.

CT was used in 55% of patients to assess the extent of ARWMC, and MRI was used in 45%. Although MRI is more sensitive than CT in detecting small punctate white-matter lesions (corresponding to grade 1 lesions of the rating scale we used), and there is inevitable variability in sensitivity to white-matter lesions between different scanners, there is good agreement between CT and MRI in detection of confluent and diffuse white-matter lesions (corresponding to grade 2 and 3 lesions of the rating scale).^34^ The inherent increased sensitivity of MRI could have led to higher ARWMC score in patients with MRI rather than CT. However, the proportion of patients who had a CT scan only in the group randomised to stenting was similar to that in the group randomised to surgery (285 of 536 *vs* 281 of 501; p=0·35) and therefore the differences in baseline imaging technique do not explain the difference between the two arms of the trial. Moreover, clinically relevant, more extensive white-matter lesions shown in our study to be a risk factor for stenting are unlikely to have been missed by using CT.

A strength of our analysis is that we used a semi-quantitative analysis of white-matter lesions using two investigators masked to clinical details who rated all images by consensus. However, because not all images were available in digital format, and most participants did not have volumetric (three-dimensional) T2-weighted or FLAIR images, we were not able to use automated or semi-automated quantitative measurements of white-matter lesion volume.

As can be expected from the pathophysiology of white-matter disease, presence of cerebral infarcts at baseline, hypertension, current smoking, and raised cholesterol concentrations at baseline were more frequent in patients with severe age-related white-matter disease. The analysis took into account these vascular risk factors and thus differences in underlying risk factor profiles in patients with high ARWMC scores compared with those with low ARWMC scores are unlikely to have introduced bias.

Our findings suggest that the presence of white-matter disease on brain imaging should be taken into account when selecting patients for carotid revascularisation (panel). Carotid artery stenting should be avoided in patients with more severe white-matter disease, but might be an acceptable alternative to carotid endarterectomy in patients with no or mild disease. Future studies of carotid revascularisation should incorporate plaque imaging and measures of cerebral haemodynamics before treatment.

Panel **Research in context**

**Systematic review**

We searched Medline for articles published in any language with the search terms “carotid stenosis”, “endarterectomy”, “stenting”, “leukoaraiosis”, and “white matter” to identify publications reporting the effect of white-matter lesions seen on brain imaging on the periprocedural risk of stroke associated from revascularisation of carotid stenosis by carotid endarterectomy or carotid artery stenting. Our last search was done on April 28, 2013. We identified only one article reporting a doubling of 30-day perioperative risk of stroke or death in patients with leukoaraiosis on brain CT treated by carotid endarterectomy in the North American Symptomatic Carotid Endarterectomy Trial (NASCET),^14^ which included 2618 patients with carotid stenosis and was completed 20 years ago. We identified no publications reporting the effect of white-matter changes on the periprocedural risk of stenting. One publication reported that in a series of 70 patients the volume of white-matter lesions correlated with the need for intraoperative shunting during carotid endarterectomy, an index of impaired cerebral haemodynamics.^30^ Another publication reported that the number of white-matter lesions before surgery was associated with the histological finding of unstable plaque in 57 patients having carotid endarterectomy for symptomatic carotid stenosis.^31^ A meta-analysis of the randomised trials comparing carotid endarterectomy with carotid artery stenting showed a higher 30 day risk of stroke after carotid artery stenting compared with carotid endarterectomy, but the effect of white-matter lesions on this comparison was not known.^5^

**Interpretation**

Our results indicate that the presence of white-matter lesions on brain imaging should be taken into account when selecting patients for carotid revascularisation. Carotid artery stenting should be avoided in patients with more extensive white-matter lesions, but might be an acceptable alternative to carotid endarterectomy in patients with less extensive lesions. Future studies of carotid revascularisation should incorporate plaque imaging and measures of cerebral haemodynamics before treatment.
