# Supplementary material for: Effect of white-matter lesions on the risk of periprocedural stroke after carotid artery stenting versus endarterectomy in the International Carotid Stenting Study (ICSS): a prespecified analysis of data from a randomised trial
Source: Lancet Neurol. 2013 Sep;12(9):866–72. doi: 10.1016/S1474-4422(13)70135-2 (PMC3744748; doi:10.1016/S1474-4422(13)70135-2)
Supplement: Supplementary appendix [file mmc1.pdf]

THE LANCET Neurology

Supplementary webappendix

This webappendix formed part of the original submission and has been peer reviewed. We post it as supplied by the authors.

Supplement to: Ederle J, Davagnanam I, van der Worp HB, et al, on behalf of the ICSS investigators. Effect of white-matter lesions on the risk of periprocedural stroke after carotid artery stenting versus endarterectomy in the International Carotid Stenting Study (ICSS): a prespecified analysis of data from a randomised trial. *Lancet Neurol* 2013; published online July 12. http://dx.doi.org/10.1016/S1474-4422(13)70135-2.

International Carotid Stenting Study (ICSS) Investigators

Steering Committee: A Algra, J Bamford (chair), J Beard, M Bland, A W Bradbury, M M Brown (chief investigator), A Clifton, P Gaines, W Hacke, A Halliday, I Malik, J L Mas, A J McGuire, P Sidhu, G Venables.

Credential committee: A Bradbury, M M Brown, A Clifton, P Gaines.

Data Monitoring Committee: R Collins, A Molyneux, R Naylor, C Warlow (chair).

Outcome Event Adjudication Committee: J M Ferro, D Thomas.

Central office staff at UCL Institute of Neurology: L H Bonati, L Coward, J Dobson (trial statistician), J Ederle, R F Featherstone (trial manager), H Tindall, D J H McCabe, A Wallis.

Participating centres by country (number of enrolled patients per centre; local investigators):

Australia

Austin Health, Heidelberg (46; M Brooks, B Chambers [principal investigator], A Chan, P Chu, D Clark, H Dewey, G Donnan, G Fell, M Hoare, M Molan, A Roberts, N Roberts).

Box Hill Hospital (Monash University), Melbourne (25; B Beiles, C Bladin [principal investigator], C Clifford, G Fell, M Grigg, G New).

Monash Medical Centre, Clayton (26; R Bell, S Bower, W Chong, M Holt, A Saunder, P G Than [principal investigator]).

Princess Alexandra Hospital, Brisbane (48; S Gett, D Leggett, T McGahan [principal investigator], J Quinn, M Ray, A Wong, P Woodruff).

Repatriation General Hospital, Daw Park, Adelaide (6; R Foreman, D Schultz [principal investigator], R Scroop, B Stanley).

Royal Melbourne Hospital, Melbourne (57; B Allard, N Atkinson, W Cambell, S Davies [principal investigator], P Field, P Milne, P Mitchell, B Tress, B Yan).

Royal Hobart Hospital, Hobart (18; A Beasley, D Dunbabin, D Stary, S Walker [principal investigator]).

Belgium

Antwerp University Hospital, Antwerp (10; P Cras, O d'Archambeau, J M H Hendriks [principal investigator], P Van Schil).

A Z St Blasius, Dendermonde (5; M Bosiers [principal investigator], K Deloose, E van Buggenhout).

A Z Sint Jan Brugge-Oostende, Campus Brugge, Brugges (18; J De Letter, V Devos, J Ghekiere, G Vanhooren [principal investigator]).

Cliniques Universitaires St Luc, Bruxelles (1; P Astarci, F Hammer, V Lacroix, A Peeters [principal investigator], R Verhelst).

Imelda Ziekenhuis, Bonheiden (3; L DeJaegher [principal investigator], A Peeters, J Verbist).

Canada

CHUM Notre-Dame Hospital, Montreal (30; J-F Blair, J L Caron, N Daneault, M-F Giroux, F Guilbert, S Lanthier, L-H Lebrun, V Oliva, J Raymond, D Roy [principal investigator], G Soulez, A Weill).

Foothills Medical Centre, Calgary (4; M Hill [principal investigator], W Hu, M Hudion, W Morrish, G Sutherland, J Wong).

Finland

Helsinki University Central Hospital, Helsinki (33; A Albäck, H Harno, P Ijäs, M Kaste [principal investigator], M Lepäntalo, S Mustanoja, T Paananen, M Porras, J Putaala, M Railo, T Sairanen, L Soinne, A Vehmas, P Vikatmaa).

ICSS Investigators (continued)

Germany

Otto von Guericke University, Magdeburg (9; M Goertler [principal investigator], Z Halloul, M Skalej).

Ireland

Beaumont Hospital, Dublin (4; P Brennan, C Kelly, A Leahy, J Moroney [principal investigator], J Thornton).

Netherlands

Academic Medical Centre, Amsterdam (56; M J W Koelemay, P J Nederkoorn [principal investigator], J A A Reekers, Y B W E M Roos).

Erasmus Medical Centre, Rotterdam (75; J M Hendriks, P J Koudstaal [principal investigator], P M T Pattynama, A van der Lugt, L C van Dijk, M R H M van Sambeek, H van Urk, H J M Verhagen).

Haga Teaching Hospitals, The Hague (45; C M A Bruijninckx, S F de Bruijn, R Keunen, B Knippenberg, A Mosch [principal investigator], F Treurniet, L van Dijk, H van Overhagen, J Wever).

Isala Klinieken, Zwolle (14; F C de Beer, J S P van den Berg [principal investigator], B A A M van Hasselt, D J Zeilstra).

Medical Centre Haaglanden, The Hague (3; J Boiten [principal investigator], J C A de Mol van Otterloo, A C de Vries, G J Lycklama a Nijeholt, B F W van der Kallen).

UMC St Radboud, Nijmegen (13; J D Blankensteijn, F E De Leeuw, L J Schultze Kool [principal investigator], J A van der Vliet).

University Medical Centre, Utrecht (270; G J de Borst, G A P de Kort, L J Kapelle [principal investigator], T H Lo, W P Th M Mali, F Moll, HB van der Worp, H Verhagen).

New Zealand

Auckland City Hospital, Auckland (40; P A Barber, R Bourchier, A Hill, A Holden, J Stewart [principal investigator]).

Norway

Rikshospitalet University Hospital, Oslo (16; S J Bakke [principal investigator], K Krohg-Sørensen, M Skjelland, B Tennøe).

Poland

Institute of Psychiatry and Neurology (2nd Department of Neurology & Department of Neuroradiology) and Medical University of Warsaw (2nd Department of General, Vascular and Oncological Surgery), Warsaw (20; P Bialek, Z Biejat, W Czepiel, A Czlonkowska [principal investigator], A Dowzenko, J Jedrzejewska, A Kobayashi, M Lelek, J Polanski).

Slovenia

University Medical Centre, Ljubljana (12; J Kirbis, Z Milosevic, B Zvan [principal investigator]).

Spain

Hospital Clinic, Barcelona (18; J Blasco, A Chamorro [principal investigator], J Macho, V Obach, V Riambau, L San Roman).

Parc Taulí Sabadell Hospital, Barcelona (33; J Branera, D Canovas [principal investigator], Jordi Estela, A Gimenez Gaibar, J Perendreu).

Sweden

Malmö University Hospital, Malmö (67; K Björses, A Gottsater [principal investigator], K Ivancev, T Maetzsch, B Sonesson).

Sodersjukhuset, Stockholm (55; B Berg, M Delle, J Formgren, P Gillgren, T-B Kall, P Konrad [principal investigator], N Nyman, R Takolander).

The Karolinska Institute, Stockholm (5; T Andersson, J Malmstedt, M Soderman, C Wahlgren, N Wahlgren [principal investigator]).

Switzerland

Centre Hospitalier Universitaire Vaudois, Lausanne (12; S Binaghi, L Hirt, P Michel [principal investigator], P Ruchat).

University Hospital Basel, Basel (94; L H Bonati, S T Engelter, F Fluri, L Guerke, A L Jacob, E Kirsch, P A Lyrer [principal investigator], E-W Radue, P Stierli, M Wasner, S Wetzel).

University Hospital of Geneva, Geneva (16; C Bonvin, A Kalangos, K Lovblad, N Murith, D Ruefenacht, R Sztajzel [principal investigator]).

UK

Addenbrookes Hospital, Cambridge (5; N Higgins, P J Kirkpatrick, P Martin [principal investigator]). K Varty.

Birmingham Heartlands Hospital, Birmingham (11; D Adam, J Bell, A W Bradbury, P Crowe, M Gannon, M J Henderson, D Sandler, R A Shinton [principal investigator], J M Scriven, T Wilmink).

Lancashire Teaching Hospitals NHS Trust, Preston (2; S D'Souza, A Egun, R Guta, S Punekar, D M Seriki [principal investigator], G Thomson).

Liverpool Royal Infirmary (21) and the Walton Centre, Liverpool (7; J A Brennan, T P Enevoldson, G Gilling-Smith [principal investigator], D A Gould, P L Harris, R G McWilliams, H-C Nasser, R White).

Manchester Royal Infirmary, Manchester (2; K G Prakash, F Serracino-Inglott, G Subramanian [principal investigator], J V Symth, M G Walker).

Newcastle Acute Hospitals NHS Foundation Trust, Newcastle upon Tyne (108; M Clarke, M Davis, S A Dixit, P Dorman [principal investigator], A Dyker, G Ford, A Golkar, R Jackson, V Jayakrishnan, D Lambert, T Lees, S Louw, S Macdonald, A D Mendelow, H Rodgers, J Rose, G Stansby, M Wyatt).

North Bristol NHS Trust, Frenchay Hospital, Bristol (13; T Baker, N Baldwin [principal investigator], L Jones, D Mitchell, E Munro, M Thornton).

Royal Free Hospital, London (1; D Baker, N Davis, G Hamilton [principal investigator], D McCabe, A Platts, J Tibballs).

Sheffield Teaching Hospitals NHS Foundation Trust, Sheffi eld (151; J Beard, T Cleveland, D Dodd, P Gaines, R Lonsdale, R Nair, A Nassef, S Nawaz, G Venables [principal investigator]).

St George's University of London and St George's NHS Healthcare Trust, London (58; A Belli, A Clifton, G Cloud, A Halliday, H Markus [principal investigator], R McFarland, R Morgan, A Pereira, A Thompson).

St Mary's Hospital, Imperial College Healthcare NHS Trust, London (13; J Chataway [principal investigator], N Cheshire, R Gibbs, M Hammady, M Jenkins, I Malik, J Wolfe).

University College London Hospitals NHS Foundation Trust, London (51; M Adiseshiah, C Bishop, S Brew, J Brookes, M M Brown [principal investigator], R Jäger, N Kitchen).

University Hospital of South Manchester, Wythenshawe, Manchester (58; R Ashleigh, S Butterfi eld, G E Gamble, C McCollum [principal investigator], A Nasim, P O'Neill, J Wong).

Western Infirmary, Glasgow (5; R D Edwards, K R Lees, A J MacKay, J Moss [principal investigator], P Rogers).
